# DrugGenEx-Net: a novel computational platform for systems pharmacology and gene expression-based drug repurposing

**DOI:** 10.1186/s12859-016-1065-y

**Published:** 2016-05-05

**Authors:** Naiem T. Issa, Jordan Kruger, Henri Wathieu, Rajarajan Raja, Stephen W. Byers, Sivanesan Dakshanamurthy

**Affiliations:** Department of Oncology, Lombardi Comprehensive Cancer Center, Georgetown University Medical Center, Washington DC, 20057 USA; Department of Biochemistry & Molecular Biology, Georgetown University, Washington DC, 20057 USA; Georgetown University Medical Center, Washington DC, 20057 USA; George Mason University, 4400 University Dr, Fairfax, VA 22030 USA

**Keywords:** DrugGenEx-NET, TMFS, Polypharmacology, Gene expression analysis, Rheumatoid arthritis, Inflammatory bowel disease, Parkinson’s disease, Alzheimer’s disease

## Abstract

**Background:**

The targeting of disease-related proteins is important for drug discovery, and yet target-based discovery has not been fruitful. Contextualizing overall biological processes is critical to formulating successful drug-disease hypotheses. Network pharmacology helps to overcome target-based bottlenecks through systems biology analytics, such as protein-protein interaction (PPI) networks and pathway regulation.

**Results:**

We present a systems polypharmacology platform entitled DrugGenEx-Net (DGE-NET). DGE-NET predicts empirical drug-target (DT) interactions, integrates interaction pairs into a multi-tiered network analysis, and ultimately predicts disease-specific drug polypharmacology through systems-based gene expression analysis. Incorporation of established biological network annotations for protein target-disease, −signaling pathway, −molecular function, and protein-protein interactions enhances predicted DT effects on disease pathophysiology. Over 50 drug-disease and 100 drug-pathway predictions are validated. For example, the predicted systems pharmacology of the cholesterol-lowering agent ezetimibe corroborates its potential carcinogenicity.

When disease-specific gene expression analysis is integrated, DGE-NET prioritizes known therapeutics/experimental drugs as well as their contra-indications. Proof-of-concept is established for immune-related rheumatoid arthritis and inflammatory bowel disease, as well as neuro-degenerative Alzheimer’s and Parkinson’s diseases.

**Conclusions:**

DGE-NET is a novel computational method that predicting drug therapeutic and counter-therapeutic indications by uniquely integrating systems pharmacology with gene expression analysis. DGE-NET correctly predicts various drug-disease indications by linking the biological activity of drugs and diseases at multiple tiers of biological action, and is therefore a useful approach to identifying drug candidates for re-purposing.

**Electronic supplementary material:**

The online version of this article (doi:10.1186/s12859-016-1065-y) contains supplementary material, which is available to authorized users.

## Background

Modern drug discovery endeavors are only rarely translated into acceptable clinical success rates [1]. Pre-clinical drug discovery initiatives have been gene-centric with a focus on finding drugs for targets of interest with high binding affinity and selectivity [2]. It is increasingly accepted, however, that disease states exhibit biological complexity, and that the gene-centric view neglects physiologic context by isolating the target in an artificial environment [3]. Furthermore, drugs arising from de novo design are likely to have many unknown targets given the limited scope of biochemical assays, thus leading to both clinical toxicity and unanticipated novel disease indications [4]. Systems pharmacology, the integration of systems biology with network pharmacology, is a mechanism-centric solution that considers the global physiological environment of disease states and allows for the discovery of drugs or combinations of drugs that may simultaneously target multiple nodes of the disease-associated network [5]. Initiatives utilizing network analysis have led to successful drug discovery efforts [6–11].

As most FDA-approved drugs are considered safe and simultaneously exhibit multi-target effects, drug repurposing is an optimal strategy for harnessing the strength of polypharmacology [12]. Current methods do not utilize high-throughput approaches to empirically determine drug-target associations and subsequently contextualize them using systems biology. Here, we have created a novel computational systems pharmacology platform, entitled DGE-NET, that: (1) accurately predicts drug-protein target interactions, (2) assesses drug effects through systems analysis of cumulative predicted targets for each drug, and (3) formulates drug-disease associations through gene expression analysis and polypharmacology.

DGE-NET was first applied to a set of 3,671 FDA approved and experimental drugs across 2,335 human protein target crystal structures for potential drug repurposing. Drugs were then associated with biological effects, which include molecular functions, signaling pathways, protein-protein interactions (PPIs) and diseases, through association with their predicted targets. Drug-biological effect predictions were validated at multiple tiers using findings in the literature and experimentally determined associations from annotated databases. Over 50 drug-disease and 100 drug-pathway associations were validated. DGE-NET also provided further evidence for unexpected toxicities, such as the potential carcinogenic properties of the cholesterol absorption blocker ezetimibe. Drug-target and drug-biological effect signatures were also statistically associated with clinical disease-relevant protein targets, PPIs, pathways, and functions obtained from differential gene expression analysis. DGE-NET incorporated a novel drug prioritization scheme that ranks drugs matched to a disease based on its polypharmacology at each tier of biological action.

For proof-of-concept, DGE-NET was applied to human-derived gene expression datasets obtained for rheumatoid arthritis (RA), inflammatory bowel disease (IBD), Alzheimer’s disease (AD), and Parkinson’s disease (PD). DGE-NET was validated by prioritizing approved drugs and biologics as well as those currently being examined repurposing, and also revealed drugs contra-indicated in those conditions, such as tetracyclines in IBD. DGE-NET is first computational platform we know of that predicts novel protein binding signatures of FDA-approved drugs and subsequently matches drug action at multiple levels of biological activity to gene expression-based characterization of disease perturbation. It stands as an effort to address the pressing need for models that account for the complexity of multi-tiered interactions for better simulations of disease states and predictive therapeutics. In summary, DGE-NET is a novel computational method for gene expression- and systems polypharmacology-driven drug repurposing.

## Methods

### Collection of FDA-approved drugs, experimental molecules, and protein target curation

Spatial Data Files (SDF) of drugs and experimental molecules containing spatial atom connectivity information were obtained from DrugBank [13], the NCGC Pharmaceutical Collection [14], FDA (www.FDA.gov), and BindingDB [15]. Energy-minimized 3D structures were prepared using Schrodinger’s LigPrep [16] algorithm at pH 7.0. Human protein crystal structures were obtained from RCSB (www.rcsb.org). Only X-ray structures with <2.5 angstrom resolution and a reference co-crystallized ligand were chosen. Protein structures were further processed to remove non-biologically relevant chains (i.e. those that do interact with the ligand), metal ions, and all heteroatoms (i.e. non-cofactors, solvent molecules). Structures were then prepared using ProteinPrep in Schrodinger to relax the structures and optimize hydrogen bonds at pH 7.0. After processing, the dataset included 3,671 drugs and 2,335 protein target crystal structures.

### Predicting Drug-Target (DT) signatures

**Fig. 1:**
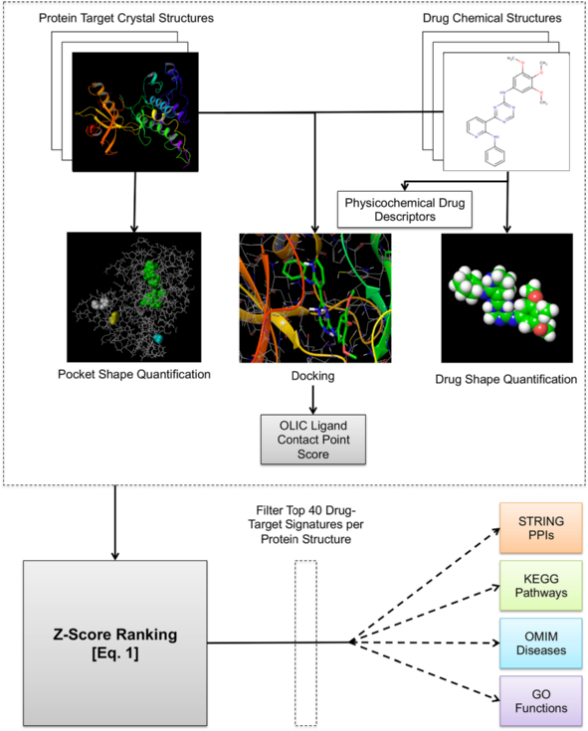
Workflow for predicting drug-target signatures and relating network pharmacology

DGE-NET utilizes a modified version of our “Train, Match, Fit and Streamline” (TMFS) method [17] for generating reliable binding signature predictions. Briefly, TMFS is a proteochemometric method that predicts the binding potential of a protein-ligand complex by integrating docking, three-dimensional shape, and ligand physicochemical descriptors (Fig. 1). GLIDE [18] was used to dock molecules into protein pockets identified by the reference ligand, and QikProp [19] was used to generate the following ligand-specific physicochemical descriptors: (1) solvent-accessible surface area, (2) volume, (3) dipole, (4) # H-bond acceptors, (5) # H-bond donors, (6) globularity, (7) ionization potential, and (8) electron affinity. Strike [20] was used to generate Tanimoto similarity coefficients to quantify the similarity of ligand physicochemical descriptors to that of the bioactive reference molecules found in the protein complex crystal structures. Ligand and pocket 3D shapes were quantified using a spherical harmonics expansion approach [21] and ligand-reference molecule/ligand-protein pocket shape similarities were quantified using a Euclidean distance metric. After docking scores, shape similarity, Euclidian distance scores, and ligand-based descriptor similarity scores were derived by the tools described above, a common scheme was used to normalize these scores, wherein each is transformed into a 0–1 range, 1 being the most favorable score present. These metrics were combined into a comprehensive Z-score that was used to rank ligands such that the top-ranking molecules are considered most likely to bind. The Z-score for a unique ligand (*l*) –protein (*p*) co-crystallized with reference ligand *r* is as follows:

1 $$ Z\left(l,r,p\right)={w}_kY\left(l,p\right)+{\displaystyle \sum_{m=1}^M\left[{w}_m{f}_m\left(l,p\right)+{w^{\hbox{'}}}_m{f^{\hbox{'}}}_m\left(l,r\right)\right]}+{\displaystyle \sum_{n=1}^N\left[{X}_n\left(l,r\right)+CS(OLIC)\right]} $$

*Y* is the normalized docking score with weight *w*_*k* = 4_. The first summation term is the normalized shape similarity score for ligand-to-protein pocket *f*_*m*_(*l*, *p*) and ligand-to-reference *f*^'^_*m*_(*l*, *r*) with weights *w*_*m* = 1_ and *w*^'^_*m* = 2_, and the second summation term corresponds to the sum of the Tanimoto similarity coefficients between the ligand and reference for physicochemical descriptors. Aforementioned weights for docking, protein shape similarity, and ligand shape similarity, respectively, were found to maximize the accuracy of TMFS in predicting top protein targets from publically available experimental data. Lastly, *CS*(*OLIC*) is a correction term based on the similarity of contact points created between the ligand and reference to the protein target. It was assumed that drugs have similar experimental activity if their interaction involves similar binding site residues and interaction patterns to that of the reference. The top 40-scoring drugs were considered as “hits” for a given protein target for subsequent network analysis. The top 40 drugs were chosen as they represent the top 1 % of all the drugs in our dataset, a fraction that is typically employed in virtual screening protocols [17].

### Relating drug-target predictions to diseases, pathways, functions, and protein-protein interactions

Predicted drug-target associations were associated with diseases, signaling pathways and molecular functions for network analysis (Fig. 1). Protein targets were cross-referenced using the unique PDB entry with UniProt [22]. Because many crystal structures may correspond to the same protein, collapsing them using UniProt reduces the total number of protein target nodes. A list of genes associated with the protein were obtained from each UniProt entry and mapped to Online Mendelian Inheritance in Man (OMIM) Morbidity Map [23] gene-disease associations, a procedure modeled after Yildirim et al. [24]. Drugs are connected to a disease via mapping of their target genes to their associated disease. Thus, a drug is connected to a disease if its predicted targets have disease genes associated with the disorder. In the DT-disease network, all disorders associated with a predicted protein target will be associated with the drug.

Disease-associated targets were also annotated with KEGG pathway [25, 26] and Gene Ontology (GO) molecular function [27, 28] information using the Database for Annotation, Visualization, and Integrated Discovery (DAVID) Functional Annotation Tool (FAT) [29, 30]. FAT was also used to annotate pathways and functions for a given drug via its predicted direct and indirect targets through protein-protein interactions using FDR <0.25. Protein-protein interactions (PPIs) were extracted from the ExPASy STRING database [31] using a confidence score cutoff of 0.95. Any PPI pairs where one of the partners did not exist in our protein target dataset were excluded. A gene list comprised of a drug’s predict direct targets as well as those targets’ interacting partners was subjected to DAVID annotation. For example, if Drug A was predicted to interact with Target A and Target B, and Target A also interacted with Protein C while Target B interacted with Protein D and Protein E, then the gene list for Drug A would consist of the following: Target A, Target B, Protein C, Protein D, and Protein E.

### Annotating disease and pathway categories

The disease categories from Medical Subject Headings (MeSH) were used for annotation of disease names corresponding to OMIM disorder entries. Approximately 93 % of the diseases were mapped to a disease category. The Comparative Toxicogenomics Database (CTD) [32] was used to map 75 % of the diseases; the remaining diseases were manually curated, with 71 % of these providing a partial or close match. Diseases that mapped to multiple disease categories were manually evaluated to determine a primary disease category. This was done by determining what the primary clinically treated category is for a disease. For example, the disease systemic lupus erythematosus is primarily an autoimmune disorder but can be considered as “skin and connective tissue” if the disease process involves the facial malar rash. Diseases in which a primary category could not be determined were categorized as multiple. Pathways were manually organized into categories based on metabolic/cellular processes and diseases as annotated by KEGG.

### Incorporation of disease gene expression data with systems pharmacology

**Fig. 2:**
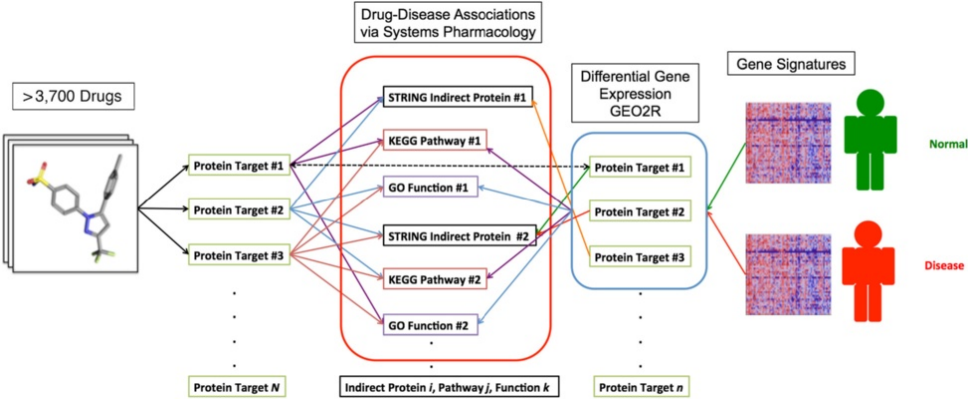
Schematic of DGE-NET used to associate drugs with diseases. Differential gene expression analysis of diseased versus non-diseased states is used to establish a disease-related gene set. DAVID and STRING analysis of this gene set provides disease-related pathways, functions, and protein-protein-interactions

A schematic of DGE-NET is illustrated in Fig. 2. Differential gene expression analysis on Gene Expression Omnibus (http://www.ncbi.nlm.nih.gov/geo/) microarray data was performed for RA (GSE55235 and GSE55457), IBD (GSE52746 and GSE11223), AD (GSE29378), and PD (GSE7621). Differentially expressed genes between normal and diseased patient biopsies with adjusted *P* values < 0.05 (using GEO2R [33]) were obtained. GEO2R is a R-based publicly accessible web tool for analyzing GEO-deposited gene expression data (http://www.ncbi.nlm.nih.gov/geo/geo2r/). The differential gene list was subjected to functional systems biology annotation as noted above. For disease sets, multiple testing correction yielded few genes having significantly differential expression. Nominal *P*-values < 0.05 were therefore used to allow for robust overrepresentation analyses. For the IBD set (normal colonic tissue control versus active IBD without anti-TNF therapy), the top 1,500 up-regulated and top 1,500 down-regulated genes were taken to create a list of 3,000 genes – the maximum number that DAVID accepts. All other datasets resulted in differential gene lists of fewer than 3,000 genes.

**Fig. 3:**
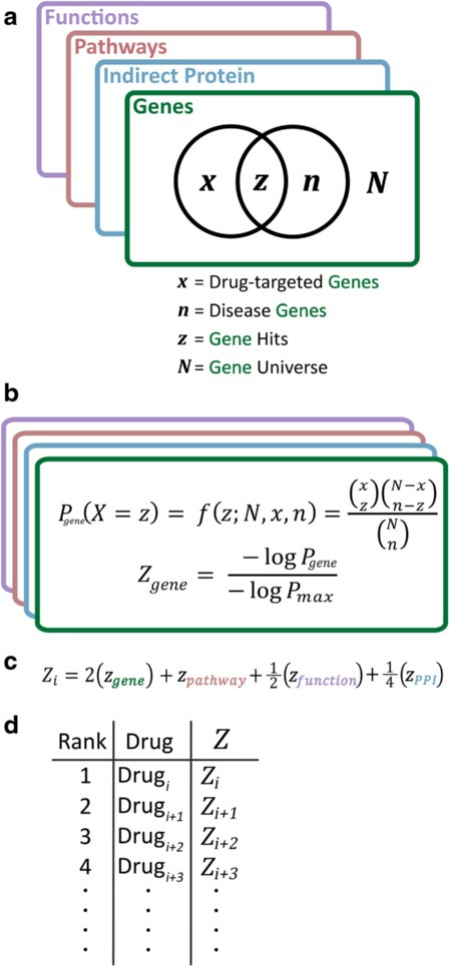
Hypergeometric test schematic for drug-disease association at each level of biological activity. Each drug is associated with a given disease at each level of biological action by the hypergeometric test. **a** Given a gene, pathway, function, or indirect protein ‘universe’, the hypergeometric test allows one to determine the probability that coincident drawings between two samples drawn from that universe is due to random chance. Therefore, the statistical significance of having hits (common items) between drug-associated biological factors and disease-associated factors is derived. **b** Computation of hypergeometric *p*-values and subsequent normalization for integration into cumulative score. **c** Computation of drug-disease association Z-score. **d** Ranking scheme by drug-disease association Z-score in descending order. That is, Zi exhibits the highest system-wide statistical association (highest-magnitude Z-score), followed by Zi + 1, Zi + 2, Zi + 3, and so forth

Using drug-target signatures from TMFS and the DGIdb [34], a comprehensive resource of experimentally determined drug-target associations curated from multiple large publically available databases, drugs were associated with diseases using the hypergeometric test (Fig. 3a) in R [35] at each of the following biological levels: direct protein targets, cell signaling pathways, molecular functions and PPIs. Drugs with *P* < 0.05 had their *P*-values log-transformed and normalized to the value of the most significantly-associated drug, resulting in values on the 0–1 unit range as illustrated in Fig. 3b. All non-significant *P*-values were automatically normalized to a value of 0. Normalization minimizes discrepancies found in the *P*-value ranges between different biological effect categories.

For each drug *i*, normalized values corresponding to each biological effect tier were used to calculate a drug-disease association Z-score used for ranking:

2 $$ {Z}_i= aA+bB+cC+dD $$

where *A*, *B*, *C*, and *D* correspond to the normalized values for drug-direct target, −pathway, −function, and –PPI associations, respectively. In illustrative Fig. 3c, A, B, C, and D correspond to zgene, zpathway, zfunction, and zPPI, respectively. Associated weights a, b, c, and d were set to the values of 2, 1, 0.5, and 0.25, respectively, as to prioritize direct binding of disease-regulated gene products with each subsequent level of activity receiving lower weights (Fig. 3c). This configuration was determined to best prioritize experimentally validated drugs for the given indication, and allowed for drugs highly associated with disease mechanisms at pathway, function, and indirect proteins levels to be recognized as candidates even when gene-level significance of association was poor. PPIs were given the least weight as many interactions tend to occur simultaneously within the diseased cell and prioritizing relevant interactions is difficult due to the simultaneous expression of thousands of proteins. Drugs are ranked in descending order by Z-score (Fig. 3d). High Z-scores indicate a drug’s potential to most significantly and simultaneously target the greatest amount of direct proteins, pathways, functions and PPIs associated with the disease. Thus, drugs with the highest Z-scores are prioritized for repurposing due to their systems-wide effects.

## Results & discussions

### Prediction of empirical drug-disease associations

**Fig. 4:**
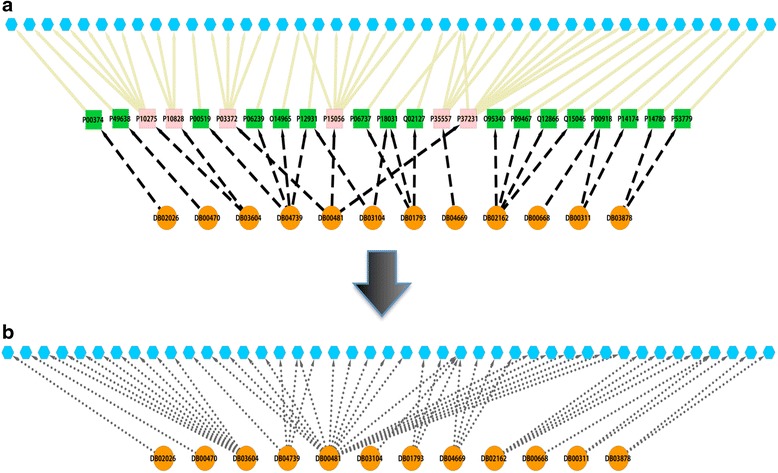
Formation of drug-target (DT) disease networks. A random sample of drugs with predicted protein targets known to be associated with a disease in OMIM were selected to illustrate the process of associating drugs with diseases. **a** Drugs (orange circle nodes) are connected using a charcoal dashed edge to predicted protein targets (square nodes); the protein targets are connected using a solid tan edge to a disease if the protein has disease genes associated with the disease. Pink nodes represent proteins associated with multiple diseases, while green nodes represent proteins associated with a single. These interactions were used to form a drug-target disease network. **b** The drugs (orange circle nodes) are connected to a disease if a predicted drug-target has disease genes associated with the disease

**Fig. 5:**
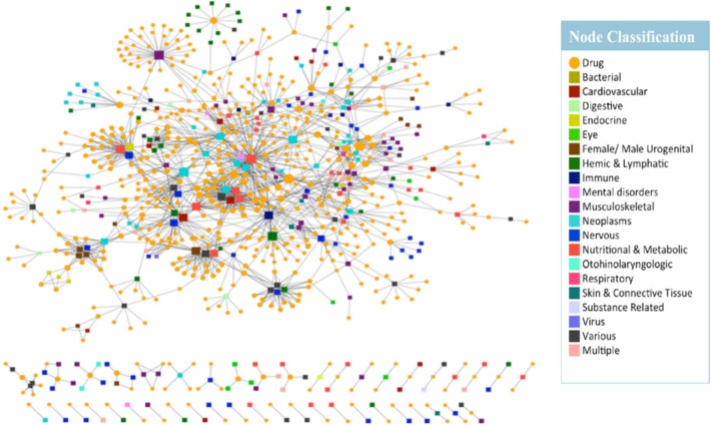
Predicted drug-target (DT) disease network. The DT disease bipartite network is generated using the top 1-ranked DT predictions and disorder-disease gene associations from OMIM. Drug nodes (circles) are connected to disease nodes (squares) if a drug is predicted to target a protein that has disease genes associated with the disease. Disease nodes are colored according to their MeSH disease category; color classification given in legend. The size of node is proportional to the number of degrees (connections)

**Fig. 6:**
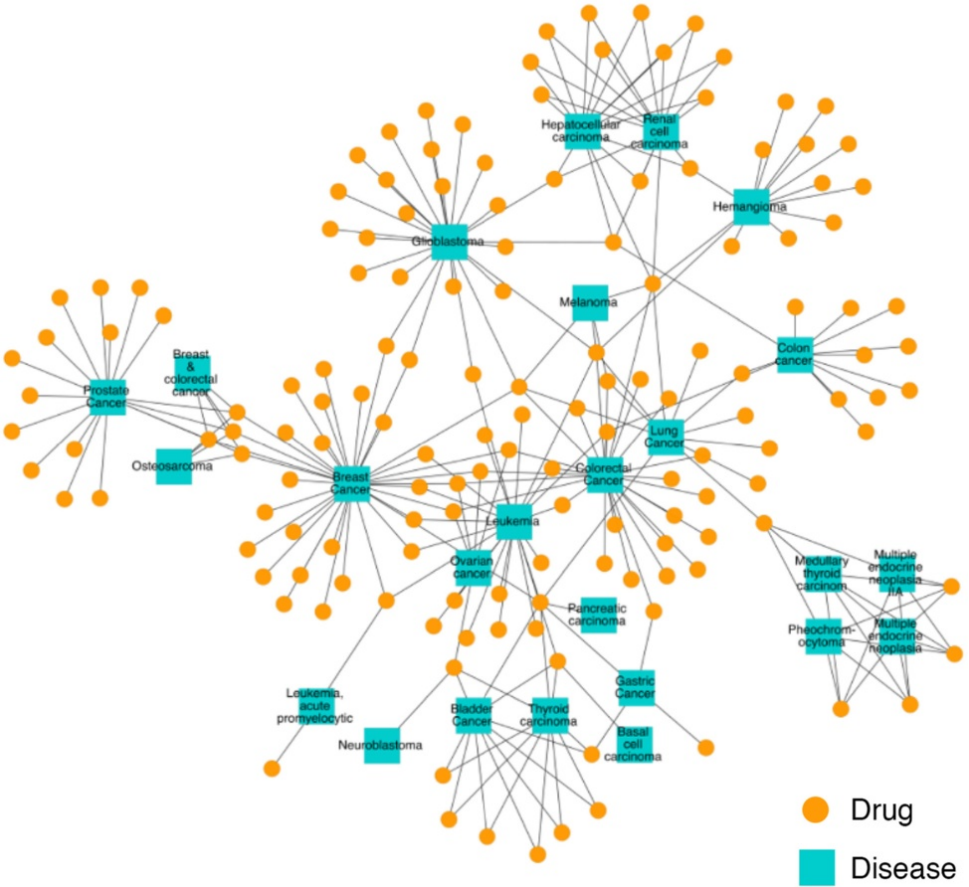
Predicted drug-cancer network from top-scoring DT interactions

DGE-NET predicted drug associations to diseases with known etiologies by way of direct gene aberrations, as annotated in OMIM (Fig. 4). The DT-disease network contains 562 drugs (only those appearing as the top 1-ranked for their respective protein target) and 296 diseases, with the largest component containing 498 drugs (Fig. 5; Additional file 1: Table S1). The neoplasm and “nutritional and metabolic” disease classes are found centrally, reflecting the large number of drugs already approved for them and a notable potential for repurposing. Given their topology in the network, associated drugs have potential polypharmacology to other disease classes. More specialized diseases tend to occupy peripheral areas of the DT-disease network, exhibiting a smaller degree of node connectivity and suggesting increasingly unique pathogenic factors. Such diseases include digestive, urogenital, “hemic and lymphatic”, and respiratory disorders. By contrast, the DT-cancer network exhibits high connectivity, with the average degree of drug nodes being 1.7 and 57 of 159 having a degree higher than 1 (Fig. 6). 26 drugs are predicted to target colorectal cancer, several of which are also predicted to target breast cancer. This is reflected in clinical practice, where several drugs are utilized across multiple cancers. The biologically sensible topology of the network provides further validation: biologically-related cancers are clustered together through their predicted drugs. For instance, the bottom right cluster contains the endocrine gland tumors medullary thyroid carcinoma, multiple endocrine neoplasia (MEN), and pheochromocytoma, whereas the unique endothelial-originating hemangioma is found isolated in the top right.

Drug-disease predictions were validated via data found in the primary literature (Additional file 2: Table S2). Out of 526 predicted drug-disease associations, 51 were validated. Full coverage is not attainable, as many drug-disease associations have not yet been examined. Nonetheless, some predicted drug-disease combinations have been well studied, such as lisinopril for diabetes-associated microvascular complications [36]. Other associations include the anti-hookworm mebendazole for hepatocellular carcinoma and the antibiotic ceftriaxone for bladder cancer. Thus, for diseases with strong single-gene known associations, DGE-NET is able to reliably predict clinically relevant drug-disease associations by forming accurate drug-target associations. These data collectively demonstrate the ability of DGE-NET to establish known and novel drug-disease associations.

### Expansion of the drug-target prediction space to systems pharmacology

**Fig. 7:**
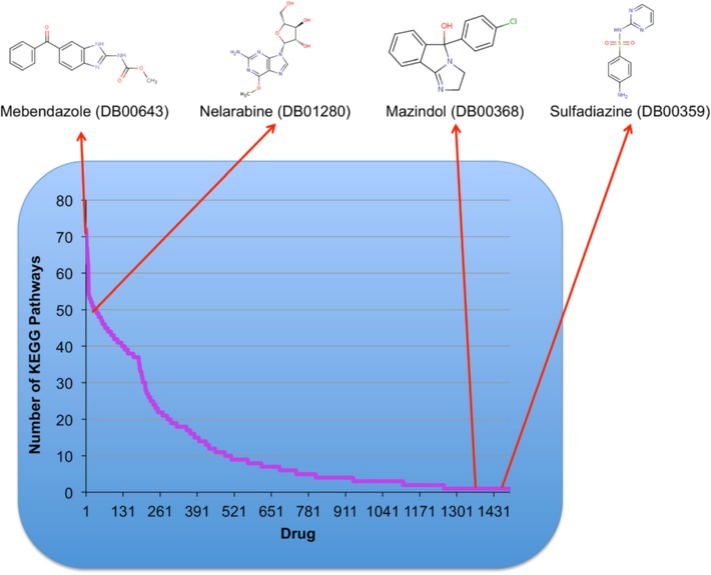
Waterfall plot for the predicted number of KEGG pathways affected by each drug

Many diseases exhibit complexity in implicating multiple perturbations rather than single deciding gene associations, and this necessitates a complex systems pharmacology perspective for clinical treatment. Drugs were therefore associated with pathways using KEGG annotations of their predicted targets. Mazindol (DB00368) and sulfadiazine (DB00359) had the least number of predicted pathways (Fig. 7). Mazindol is a tricyclic anorexigenic known to affect the noradrenergic, dopaminergic and serotonergic pathways (KEGG Drug D00367). Sulfadiazine is a sulfonamide used to treat bacterial infections by specifically inhibiting the folate biosynthesis pathway (KEGG Drug D00587). DGE-NET was able to recapitulate their specificity for those pathways. Alternatively, kinase inhibitors and nucleoside analogs such as nelarabine (DB01280) disrupt multiple pathways (Fig. 7). The KEGG Drug corpus was also used to validate 103 drug-pathway associations across 59 drugs (Table 1). Thus, DGE-NET is able to reliably associate drugs with biological pathways important in disease processes.

**Table 1:** Validations of predicted drug-pathway associations via the KEGG Drug database

Drug	KEGG Drug ID	KEGG Pathway
Acetohexamide	D00219	Type II diabetes mellitus
Aripiprazole	D01164	Gap junction
Bezafibrate	D01366	Adipocytokine signaling pathway
Bicalutamide	D00961	Pathways in cancer, Prostate cancer
Candesartan	D00626	Vascular smooth muscle contraction
Carvedilol	D00255	Vascular smooth muscle contraction
Celecoxib	D00567	VEGF signaling pathway
Cilostazol	D01896	Insulin signaling pathway
Clozapine	D00283	Gap junction
Conivaptan	D01236	Vascular smooth muscle contraction
Danazol	D00289	Oocyet meiosis, Progesterone-mediated oocyte maturation, Pathways in cancer
Dasatinib	D03658	MAPK signaling pathway, ErbB signaling pathway, Cytokine-cytokine receptor interaction, VEGF signaling pathway, Pathways in cancer, Chronic myeloid leukemia
Diflunisal	D00130	VEGF signaling pathway
Domperidone	D01745	Gap junction
Droperidol	D00308	Gap junction
Drospirenone	D03917	Aldosterone-regulated sodium transport
Dydrogesterone	D01217	Oocyte meiosis, Progesterone-mediated oocyte meiosis
Eltrombopag	D03978	Cytokine-cytokine receptor interaction, Jak-STAT signaling pathway
Epoprostenol	D00106	Vascular smooth muscle contraction
Eprosartan	D04040	Vascular smooth muscle contraction
Erlotinib	D07907	MAPK signaling pathway, ErbB signaling pathway, Cytokine-cytokine receptor interaction, Pathways in cancer, Pancreatic cancer, Non-small cell lung cancer
Fenofibrate	D00565	Adipocytokine signaling pathway
Floxuridine	D04197	Pyrimidine metabolism
Flupenthixol	D01044	Gap
Flurbiprofen	D00330	VEGF signaling pathway
Flutamide	D00586	Pathways in cancer, Prostate cancer
Gemcitabine	D02368	Purine metabolism, Pyrimidine metabolism
Gliclazide	D01599	Type II diabetes mellitus
Glipizide	D00335	Type II diabetes mellitus
Haloperidol	D00136	Gap junction
Imatinib	D01441	MAPK signaling pathway, Cytokine-cytokine receptor interaction, Hematopoietic cell lineage, Pathways in cancer, Chronic myeloid leukemia
Indacaterol	D09318	Endocytosis
Indomethacin	D00141	VEGF signaling pathway
Ketoprofen	D00132	VEGF signaling pathway
Lapatinib	D04024	MAPK signaling pathway, ErbB signaling pathway, Cytokine-cytokine receptor pathway, Pathways in cancer
Levonorgestrel	D00950	Oocyte meiosis, Progesterone-mediated oocyte maturation
Losartan	D08146	Vascular smooth muscle contraction
Methysergide	D02357	Gap junction
Milrinone	D00417	Progesterone-mediated oocyte maturation
Mitiglinide	D01854	Type II diabetes mellitus
Naproxen	D00118	VEGF signaling pathway
Nilutamide	D00965	Pathways in cancer, Prostate cancer
Norethindrone	D00182	Oocyte meiosis, Progesterone-mediated oocyte maturation
Olmesartan	D01204	Vascular smooth muscle contraction
Oxaprozin	D00463	VEGF signaling pathway
Piroxicam	D00127	VEGF signaling pathway
Progesterone	D00066	Oocyte meiosis, Progesterone-mediated oocyte maturation
Propericiazine	D01485	Gap junction
Regadenoson	D05711	Vascular smooth muscle contraction
Risperidone	D00426	Vascular smooth muscle contraction, Gap junction
Salsalate	D00428	VEGF signaling pathway
Silodosin	D01965	Vascular smooth muscle contraction
Sorafenib	D08524	MAPK signaling pathway, ErbB signaling pathway, Cytokine-cytokine receptor interaction, Chemokine signaling pathway, mTOR signaling pathway, VEGF signaling pathway, Natural killer cell mediated cytotoxicity, Pathways in cancer, Renal cell carcinoma
Sulindac	D00120	VEGF signaling pathway
Sunitinib	D06402	MAPK signaling pathway, Cytokine-cytokine receptor interaction, VEGF signaling pathway, Pathways in cancer
Telmisartan	D00627	Vascular smooth muscle contraction
Testosterone	D00075	Pathways in cancer, Prostate cancer
Vandetanib	D06407	MAPK signaling pathway, ErbB signaling pathway, Cytokine-cytokine receptor interaction, VEGF signaling pathway, Pathways in cancer

**Fig. 8:**
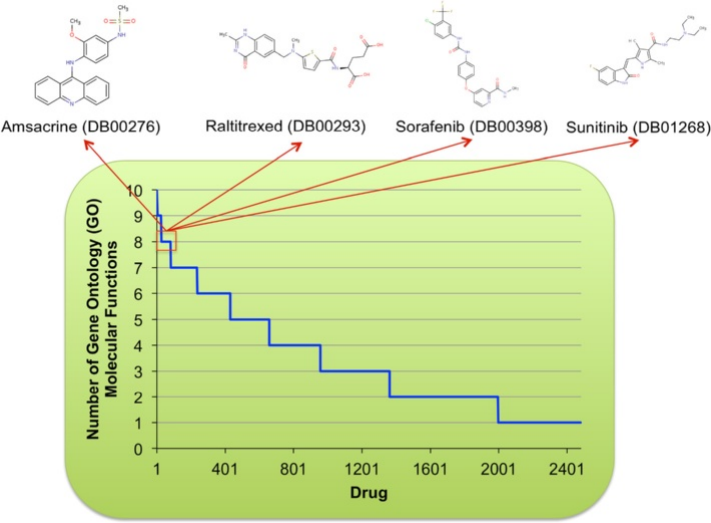
Waterfall plot for the predicted number of GO molecular functions affected by each drug. Inset highlights four anti-neoplastic drugs predicted to disrupt the greatest number of functions from the anti-neoplastic drug class

DGE-NET also related predicted DT signatures to molecular functions (Fig. 7). Deferasirox, an iron chelator, was predicted to affect the greatest number of molecular functions. According to the Institute for Safe Practices, deferasirox was the second most suspected drug in reported patient deaths [37]. This may be due to its potential to disrupt many molecular functions as predicted by DGE-NET. Anti-neoplastic drugs were also predicted to alter a large number of functions (Fig. 8). This reflects their polypharmacology as a class of drugs, as they are designed to affect cell signaling and growth through multiple mechanisms. As a result, these drugs also exhibit high toxicity. Such analysis of molecular function can have the advantage of identifying broad- or specific-acting drugs for enriched clinical efficacy or minimized toxicity.

**Fig. 9:**
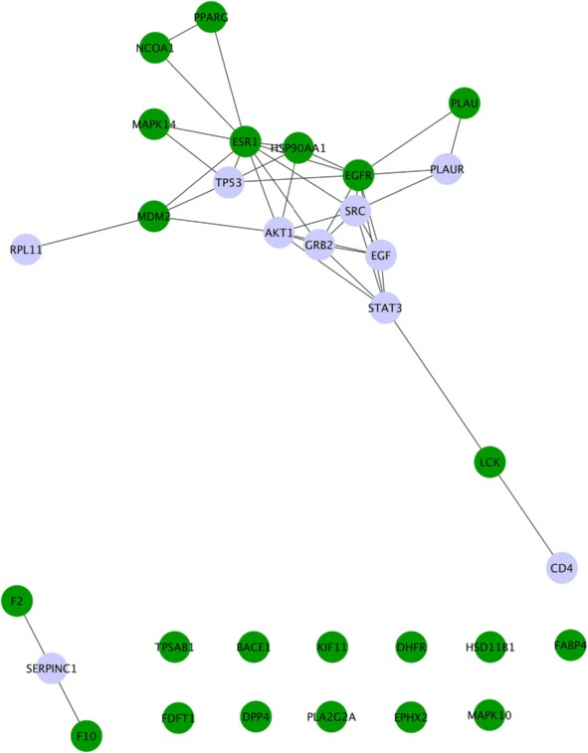
Ezetimibe protein-protein interaction (PPI) network. Direct targets (green nodes) predicted for ezetimibe from TMFS were used to establish interactions between direct targets as well as indirect targets (light purple nodes) using the ExPASy STRING database with a confidence score cutoff greater than 0.95

The incorporation of protein-protein interactions (PPIs) further increased the robustness of DGE-NET, providing insight into unexpected biological similarities among drugs. For example, fluoxymesterone (DB01185) and amscarine (DB00276) are chemically and structurally unrelated. However, our method predicted that they would bind androgen receptor and B-Raf, respectively, both of which interact with MAPK1. It is through the PPI with MAPK1 that these drugs link to pathways in cancer (KEGG hsa:05200). Other drug-PPI validations are listed in Table 2 [38–48]. To highlight the importance of PPIs in attaining a mechanistic understanding of drug effects, we specifically assessed the predicted effects of ezetimibe (Fig. 9; Additional file 3: Table S3). Ezetimibe (DB00983) is a cholesterol-lowering drug used for improving cardiovascular health and has also been associated with increased incidence of cancer [49, 50]. PPIs derived from predicted targets for ezetimibe are highly clustered, indicating that the affected biological space is tightly coordinated through those targets and greatly perturbed by the actions of ezetimibe (Fig. 9). These clustered interacting targets are mainly involved in cell growth, differentiation and signal transduction. Functional annotation using both direct and indirect ezetimibe targets implicates pathways and functions involved in carcinogenesis (Additional file 3: Table S3). Thus, the present DGE-NET prediction of ezetimibe’s pro-tumorigenic effects warrants further investigation.

**Table 2:** Validations of predicted drug-PPI interactions

Drug Name	Protein #1 (direct binding partner)	Protein #2 (PPI)	Reference
Bicalutamide	ABL1	CASP9	Danquah et al. Pharm Res. 26(9):2081–92. (2009) [38]
	ABL1	CCNA2	Katayama et al. Int J Oncol. 36(3):553–62. (2010) [39]
	ABL1	MAPK11	Malinowska et al. Endocr Relat Cancer. 16:155–169. (2009) [40]
Cladribine	ADA	DCK	Sasvári-Székely et al. Biochem Pharmacol. 56(9):1175–1179. (1998) [41]
Chlordiazepoxide	AKT1	NR3C1	Curtin et al. Brain Behav, Immun. 23(4): 535–547. (2009) [42]
Progeterone	AR	F2	Oger et al. Arterioscler Thromb Vasc Biol. 23:1671–1676. (2003) [43]
Cyproterone	AR	CASP3	Eckle et al. Toxicol Pathol. 32:9–15. (2004) [44]
	AR	NR3C1	Honer et al. Mol Pharmacol. 63(5):1012–1020. (2003) [45]
Telmisasrtan	BCL2	IL2	Syrbe et al. Hypertens Res. 30(6):521–527. (2007) [46]
Sorafenib	BRAF	PRKCQ	Jane et al. J Pharmacol Exp Ther. 319(3):1070–1080. (2006) [47]
Methotrexate	DHFR	CDK2	Maddika et al. J Cell Sci. 121:979–988. (2008) [48]

### Incorporation of autoimmune disease-related gene expression data for polypharmacology-driven drug repurposing

Autoimmune diseases are systemic or local pro-inflammatory pathologies with multiple etiologies. Current therapeutics such as corticosteroids, methotrexate and anti-TNF biologics focus on regulating inflammation, and immunosuppression. In addition to acting non-specifically these medications do not address the full extent of effector tissue pathobiology. A treatment approach rooted in polypharmacology may be more efficacious and offers the potential for limiting side effects. For proof-of-concept, we apply DGE-NET as a gene expression-based polypharmacology prediction method (Fig. 2) for rheumatoid arthritis and inflammatory bowel disease.

Rheumatoid arthritis (RA) is a painful multi-joint destructive disease. Joint synovium, usually 1–2 cells thick, becomes inflamed and reaches multicellular thickness due to infiltration of immune effector cells and activation and subsequent proliferation of fibroblast-like synoviocytes (FLS). Cellular molecular cross-talk, infiltration and proliferation lead to pannus formation, which acts analogously to an invasive tumor and causes joint destruction. As FLS cells are critical mediators of RA, we applied our method using differentially expressed genes when comparing activated FLS cells from RA patients and quiescent FLS cells from non-RA patients (GSE55235 and GSE55457). A consensus drug list was constructed by combining the top 100 (~ Top 10 % of total drug database) predicted drugs for each study and extracting those that are present in one or both lists, ranked by mean association Z-score (Additional file 4: Table S4). Shown in Table 3 are those drugs from the consensus drug list that are currently used for RA, or have been found to be potentially useful in the clinic [51–54, 97–103]. Drugs currently used in the clinic were recapitulated in our list, such as anti-TNF biologics adalimumab and etanercept, as well as the NSAID sulindac. Non-approved drugs currently being studied for RA also appeared. These include kinase inhibitors such as alvocidib [51] and sunitinib [52], the topoisomerase inhibitor karenitecin [53], and the chloroquine-related compound amodiaquine [54]. Predicted RA indication for these drugs, which are generally anti-cancer agents, illustrates an important mechanistic underpinning of RA with respect to FLS cells in that activated FLS mimic cancer cell progression [55]. Regardless of the activating stimulus (e.g. TNF-α), our polypharmacological method focuses on downstream gene expression, signaling, and functional effects in activated FLS cells. This highlights the cancer-like mechanisms of pathogenesis and prioritizes those drugs that are able to simultaneously disrupt the greatest number of those mechanisms. In addition, because antibodies have single-target effects, we were surprised by their predicted indications for RA. However, if that target has many pathology-related pleiotropic downstream effects, such as TNF-α, then such drugs would be prioritized due to the pathway and function terms in our equation. Thus, DGE-NET is capable of making important polypharmacological associations beyond immediate gene targets.

**Table 3:** Validations of predicted drug indications for RA and IBD from consensus drug lists, ordered by drug list ranking

Rheumatoid Arthritis (RA)	Reference for Validation	Inflammatory Bowel Disease (IBD)	Reference for Validation
Alvocidib	Sekine et al. J Immunol. 180(3):1954–1961 (2008) [51]	Sulfasalazine	Klotz et al. N Engl J Med. 303(26):1499–1502 (1980) [105]
Karenitecin	Liu et al. Med Res Rev. 35(4):753-89 (2015) [53]	Olsalazine	Baumgart et al. Lancet. 369(9573):1641–1657 (2007) [106]
Sulindac	Brogden et al. Drugs. 16(2):97–114 (1978) [97]	Tetomilast	Keshavarzian et al. Expert Opin Investig Drugs. 16(9):1489–1506 (2007) [107]
Sunitinib	Fuyura et al. Mod Rheumatol. 24(3):487–491 (2013) [52]	Inosine	Mabley et al. Am J Physiol Gastrointest Liver Physiol. 284(1):G138-G144 (2003) [108]
INCB28050	Taylor et al. Ann Rheum Dis. 73:A31 (2014) [99]	Thioproperazine	Lechin et al. J Clin Gastroenterol. 4(5):445–450 (1982) [56]
Amodiaquine	Kersley et al. Lancet. 2(7108):886–888 (1959) [54]	Etoricoxib	El Miedany et al. Am J Gastroenterol. 101(2):311–317 (2006) [109]
Raltitrexed	van der Heijden et al. Scand J Rheumatol. 43(1):9–16 (2014) [100]	Balsalazide	Carter et al. Gut. 53(Suppl 5):V1-V16 (2004) [110]
BIRB 796	Page et al. Arthritis Rheum. 62(11):3221–3231 (2010) [101]	Thalidomide	Gerich et al. Ailment Pharmacol Ther. 41(5):429–437 (2015) [59]
Adalimumab	Weinblatt et al. Arthritis Rheum. 48(1):35–45 (2003) [102]	Rosiglitazone	Ramakers et al. J Clin Immunol. 27(3):275–283 (2007) [57]
Etanercept	Moreland et al. Ann Intern Med. 130(6):478–486 (1999) [103]	Irbesartan	Ray et al. Gut. 62(S1):A525-A525 (2013) [60]
Minocycline	O’Dell et al. Arthritis Rheum. 40(5):842–848 (1997) [104]	Chloroquine	Nagar et al. Int Immunopharmacol. 21(2):328–335 (2014) [111]

DGE-NET also predicted drugs for inflammatory bowel disease (IBD), also a multi-etiological immune-related collection of disorders. Differential gene expression analysis was performed by, comparing normal and inflamed bowel tissues (GSE52746 and GSE11223). Like the RA dataset, a consensus list of the Top 100 drugs obtained from each IBD study was constructed (Additional file 5: Table S5), and therapeutic validations from this list are recapitulated in Table 3 [56–60, 104–110]. Our method predicted the known IBD drug sulfasalazine, serving as an important litmus. Other predicted drugs that are promising in experimental settings and from diverse chemical classes include the anti-psychotic thioproperazine [56], the anti-diabetic thiazlidinedione rosiglitazone [57], the leukotriene receptor antagonist tetomilast [58], and thalidomide [59]. Interestingly, DGE-NET predicted the angiotensin receptor blocker (ARB) irbesartan as potential therapy. A recent preliminary study implicates the role of angiotensin receptors in intestinal fibrosis in Crohn’s disease [60], a type of IBD, but greater investigation is needed.

In addition to recapitulating known drug associations, we predicted the drugs topotecan and mebendazole for repurposing to rheumatoid arthritis. Topotecan is a DNA topoisomerase 1 (Top1) inhibitor used for NSCLC cancer and has been given both orally and intravenously. Topoisomerases have been implicated in rheumatoid arthritis etiology [61], and the established Top1 inhibitor camptothecin (CPT) has been shown to be effective in a murine collagen-induced RA model [62]. Koo et al. developed a novel nanocarrier for CPT called CPT-SSM-VIP, which denotes micelles to overcome solubility issues and vasoactive intestinal peptide (VIP) for active targeting. As CPT provides evidence for Top1 inhibition in RA, we also pursued topotecan. Although it can be inferred that topotecan could be an effective anti-arthritic via topoisomerase, many other unreported targets were predicted for topotecan that could mediate potential efficacy. These include multiple tyrosine-protein kinases (BTK, CSK, LCK, TTK, ITK, LYN), non-tyrosine kinases (AURK1, PIK3CG), as well as cyclin A2. Mebendazole is an anti-hookworm tubulin inhibitor with anti-cancer potential through mammalian crossover tubulin [63] and kinase inhibition [64]. We previously predicted many novel protein kinase targets for mebendazole [17]. Kinase inhibition is a sought after therapeutic strategy for rheumatoid arthritis, especially as non-biologic treatment alternatives and for methotrexate-resistant cases [65–67]. Inhibitors of spleen tyrosine kinase (Syk) and Janus kinases (Jaks) have shown short-term efficacy, but other kinases inhibitors with good long-term effect profiles may also exist. Other kinases implicated in RA pathogenesis include aurora kinases [68] and cyclin-dependent kinases (CDKs) [69]. Mebendazole may serve as a good non-biologic disease-modifying antirheumatic drug (DMARD) given its historic use, low toxicity profile, and its effect on multiple kinases.

In another proof-of-concept, we applied DGE-NET to two neurodegenerative disorders, Alzheimer’s disease (AD) and Parkinson’s disease (PD). Table 4 summarizes those drugs predicted to be in the top 50 for AD and PD by DGE-NET that are currently validated for standard or potential therapeutic use [70–86]. The complete top 50 predicted drugs for these diseases and their validations are found in Additional file 6: Table S6. Others listed are currently undergoing pre-clinical or clinical investigation. Of note is that memantine, an approved drug for AD, appears beyond the top 50 but within the top 500. This drug exhibits less polypharmacology but is still effective given the importance of its direct targets and pathways for AD disease processes (i.e. NMDA receptor antagonism reducing glutamate excitotoxicity of neurons [87]). Thus, it can be hypothesized thatdrugs found higher up in the rank list may be more effective than the current clinical standards of care as those drugs theoretically alter a greater proportion of disease-associated protein targets and biological effects simultaneously.

**Table 4:** Validations of top 50 predicted drug indications for AD and PD, ordered by ranking

Alzheimer’s Disease (AD)	Reference for Validation	Parkinson’s Disease (PD)	Reference for Validation
Rasagiline	Weinreb et al. Neurotherapeutics. (6)1:163–74. (2009) [70]	Dextroamphetamine	Parkes et al. J Neurol Neurosurg Psychiatry. 38(3):232–7 (1975) [83]
Interferons	Grimaldi et al. J Neuroinflammation. 11:30 (2014) [71]	Orphenadrine	Bersani et al. Clin Neuropharmacol. 13(6):500–6 (1990) [84]
Calcium	Woods et al. Adv Exp Med Biol. 740:1193–217 (2012) [72]	Quinacrine	Tariq et al. Brain Res Bull. 54(1):77–82 (2001) [85]
Dovitinib	Li et al. Medical Hypotheses. (80)4:341–44. (2013) [73]	Atomoxetine	Weintraub et al. Neurology. 75(5):448–55 (2010) [86]
Somatropin Recombinant	Ling et al. Growth Horm IGF Res. (17)4:336–41 (2007) [74]		
Aripiprazole	De Deyn et al. Expert Opin. Pharmacother. (14)4:459–74 (2013) [75]		
Clozapine	Tariot et al. Clin Geriatr Med. (17)2:359–76 (2001) [76]		
Quercetin	Ansari et al. J Nutr Biochem. 20(4):269–75 (2009) [77]		
Flavopiridol	Pallàs et al. Med Hypotheses. 64(1):120–3 (2005) [78]		
Sunitinib	Grammas et al. J Alzheimers Dis. 40(3):619–30 (2014) [79]		
Risperidone	Katz et al. Int J Geriatr Psychiatry. (60)2:107–15 (2007) [80]		
Genistein	Valles et al. Brain Res. 1312:138–44 (2010) [81]		
Dasatinib	Dhawan et al. J Neuroinflammation. 9:117 (2012) [82]		

**Fig. 10:**
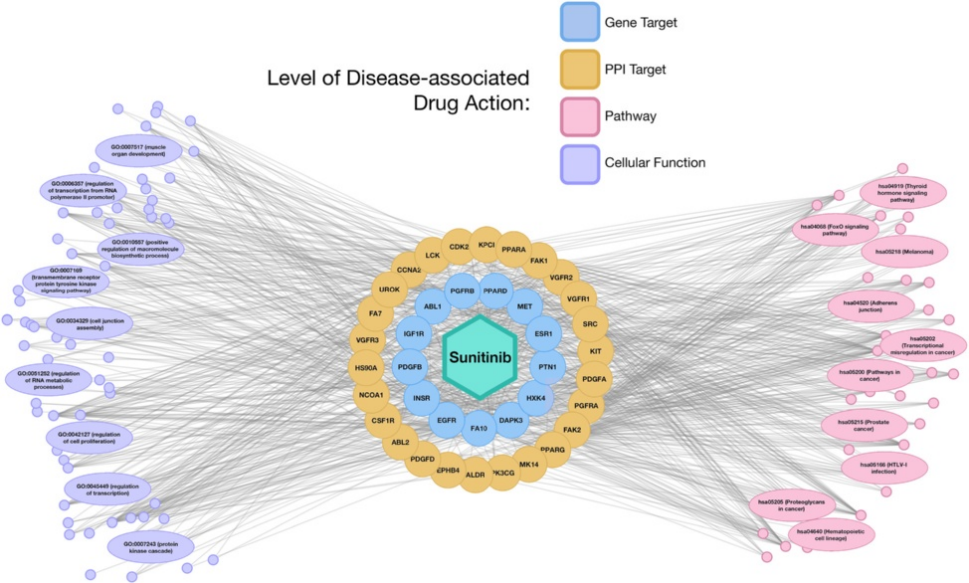
Predicted sunitinib drug action network on AD. Direct protein targets predicted by DGE-NET for sunitinib that are also significantly AD-modulated are in large orange and blue circles. Blue circles are genes overexpressed in AD with statistical significance, while orange circles are protein partners of those genes. Pink circles are KEGG pathways, and purple circles are GO cellular functions, enriched at *p*-value < .01 in the up-regulated genes of AD. The top 10 significantly enriched cellular functions and pathways are detailed in large ovals

Sunitinib has been identified as a lead candidate having the potential to mitigate the development of oxidant injury to endothelial cells associated with AD [79]. Sunitinib could affect the vascular activation mechanisms of pathogenesis in AD by reducing the expression of amyloid beta, thrombin, tumor necrosis factor alpha, interleukin-1 beta, interleukin-6, and matrix metalloproteinase 9, and other factors associated with neurodegenerative disorders [79, 88, 89]. This anti-angiogenic property has been previously shown to be a major component of the anti-cancer acitivity of sunitinib [90]. Figure 10 illustrates the polypharmacology of sunitinib, at each level of biological activity, predicted by DGE-NET to coincide with significantly AD-associated factors. Single-agent or combination therapies that exploit multiple aspects of disease process are assumed to be efficacious, requiring lower dosages than current therapies and reducing the likelihood of resistance.

In addition to therapeutic drug repurposing candidates, DGE-NET reported drugs that are known to be contra-indicated for their respective diseases. Minocycline and tretinoin, both of which are used to treat acne, may have IBD toxicity. Minocycline is a tetracycline antimicrobial with a potential association with IBD (Additional file 5: Table S5) [91]. Tretinoin is a topical retinoid that is structurally related to isotretinoin, an oral medication used for more severe acne. While tretinoin itself is safe, isotretinoin has been implicated in causing IBD (Additional file 5: Table S5) [92], though this finding is controversial. It could be extrapolated that if tretinoin was given orally and at higher doses that IBD may be a consequence. Others include methysergide, a prophylactic drug that is contra-indicated for RA and other collagen diseases (Additional file 4: Table S4) [93], indomethacin, a non-selective non-steroidal anti-inflammatory drug known to exacerbate IBD (Additional file 5: Table S5) [94, 95], quetiapine, an atypical antipsychotic associated with increased cognitive decline in AD (Additional file 6: Table S6) [96], and methamphetamine, which has been linked with an increased risk of PD (Additional file 6: Table S6), [97]. The appearance of these drugs is likely due to DGE-NET not discriminating between agonistic and antagonistic effects of drugs but rather forming non-directional drug-target-effect associations. Counter-therapeutic drug actions are therefore incorporated, so long as they correspond with disease-associated biological activity.

## Conclusions

DGE-NET is able to predict drug-target interactions and contextualize their biological effects at the levels of protein-protein interactions, biological pathways, and molecular functions. It further integrates gene expression signatures for identification of systems-based disease-relevant targets and prioritization of drugs that exhibit a desired polypharmacology. DGE-NET recapitulated known therapeutic and contraindicated drugs for rheumatoid arthritis and inflammatory bowel disease and led to the identification of mebendazole as drug repurposing candidate for rheumatoid arthritis. Its ability to do so can also be extended to other small molecules with the potential to act as endogenous drugs to alter physiology, such as metabolites. We are currently pursuing the application of DGE-NET to cancer-associated metabolites to potentially explain the mechanisms behind metabolite-disease phenotypic associations. DGE-NET ultimately assists in the formulation of drug-disease hypotheses poised for clinical success.

Differential gene expression analysis is one way of assessing disease pathogenesis to find therapeutic targets. DGE-NET is the first computational tool that associates drugs with diseases through multiple tiers of systems biology obtained via gene expression analysis. This not only aids in finding effective drugs but helps bypass issues that arise from traditional gene sequencing approaches such as un-actionable mutations in single nucleotide polymorphisms, which is currently an important limitation in oncology. Importantly, DGE-NET in its current form does not differentiate agonist or antagonist effects of drugs. The next iteration will include this improvement so that DGE-NET can better discriminate between therapeutic agents and drugs that are contraindicated.

## Availability of data and materials

Because DGE-NET is applied to publicly available data, the authors have provided a tutorial which describes the stepwise implementation of DGE-NET, in Additional file 7.

## Additional files

Additional file 1: Table S1. Predicted drug-target-disease associations using OMIM. For each human protein target crystal structure, the top 40-ranked drugs were associated with a disease through their predicted target. (XLSX 464 kb) Additional file 2: Table S2. Validations of predicted drug-disease associations from the literature. (XLS 38 kb) Additional file 3: Table S3. Predicted systems pharmacology of ezetimibe. Targets predicted to directly associate with ezetimibe and their interacting protein partners (confidence score cutoff greater than 0.95) were used to infer pathways and molecular functions (FDR < 0.25) that could be perturbed by ezetimibe. (XLS 42 kb) Additional file 4: Table S4. Consensus drug rank list for Rheumatoid Arthritis. (XLS 47 kb) Additional file 5: Table S5. Consensus drug rank list for Inflammatory Bowel Disease. (XLS 51 kb) Additional file 6: Table S6. Top 50 predicted drugs and validations for Alzheimer’s Disease and Parkinson’s Disease. (XLS 39 kb) Additional file 7: Tutorial outlining the manual implementation of the DrugGenEx-NET methodology. (DOCX 14 kb)

## Abbreviations

AD: Alzheimer’s disease
CDK: Cyclin-dependent kinase
CPT: Camptothecin
CTD: Comparative toxicogenomics database
DAVID: Database for annotation, visualization, and integrated discovery
DGE-NET: DrugGenEx-Net
DMARD: Disease-modifying antirheumatic drug
DT: Drug-target
FAT: Functional annotation tool
FLS: Fibroblast-like synoviocytes
IBD: Inflammatory bowel disease
MEN: Multiple endocrine neoplasia
MeSH: Medical subject headings
OMIM: Online mendelian inheritance in man
PD: Parkinson’s disease
PPI: Protein-protein interactions
RA: Rheumatoid arthritis
SDF: Spatial data file
TMFS: Train, match, fit, streamline
VIP: Vasoactive intestinal peptide
