# Supplementary material for: DrugGenEx-Net: a novel computational platform for systems pharmacology and gene expression-based drug repurposing
Source: BMC Bioinformatics. 2016 May 5;17:202. doi: 10.1186/s12859-016-1065-y (PMC4857427; doi:10.1186/s12859-016-1065-y)
Supplement: Additional file 7: — Tutorial outlining the manual implementation of the DrugGenEx-NET methodology. (DOCX 14 kb) [file 12859_2016_1065_MOESM7_ESM.docx]

**Tutorial for the implementation of DrugGenEx-NET**

1. Collect drug-target associations from DGIdb (http://dgidb.genome.wustl.edu/downloads), converting all gene symbols to Uniprot Accession ID (http://www.uniprot.org/), being sure to name compounds consistently or assigning them distinct IDs.
2. Obtain gene members for all KEGG pathways and GO functions from the DAVID database (https://david.ncifcrf.gov/), resulting in a set of gene-pathway associations and gene-function associations. Preserve KEGG and GO IDs, but convert all gene symbols to Uniprot Accession ID.
3. By way of association with its protein targets, assign perturbed pathways and functions to each drug, resulting drug-pathway and drug-function association datasets.
4. Download all protein-protein interaction data from the STRING database (http://string-db.org/cgi/download.pl), and extract interactions with a confidence cutoff of > .7, converting all protein IDs to Uniprot Accession ID.
5. Record the dataset series ID for the Gene Expression Omnibus (http://www.ncbi.nlm.nih.gov/geo/) microarray data corresponding to the disease or condition of interest. This series ID will be required for differential gene expression analysis.
6. Upon converting the GEO gene probe set of the microarray platform in question to Uniprot IDs, narrow down drug-target, drug-pathway, drug-function, and protein-protein interactions to only those which are derived from proteins matching the gene probes of the platform. This ensures that the researcher is working properly within the defined gene space universe.
7. Utilize the GEO2R online tool (http://www.ncbi.nlm.nih.gov/geo/geo2r/) to perform differential gene expression analysis and determine differentially expressed genes between disease and normal samples at P-value <.05. If more than 1,500 genes are significantly up- or down-regulated, extract only the top 1,500 genes ordered by fold change, for a maximum of 3,000 total differentially expressed genes. Combine all significantly disease-modulated genes and convert symbols to Uniprot Accession ID.
8. To obtain indirect proteins associated with the disease condition, use the protein-protein interaction data curated above and collect all proteins associated with disease-regulated genes.
9. To perform functional enrichment of the gene list, paste the gene list to the DAVID functional annotation tool (https://david.ncifcrf.gov/summary.jsp) and follow the required steps. Select “Gene List” as the gene type. Upon submitting the list, select “homo sapiens” as the species, and open “Gene_Ontology” and “Pathways” under the Annotation Summary Results. Download the corresponding charts, and collect only those functions and pathways overrepresented with a significance of P-value < .05.
10. Using the dhyper function in the R environment (https://www.r-project.org/), for a given drug-disease association, calculate the statistical significance of coincidence between disease-regulated and drug-perturbed factors at each level of biological activity: direct proteins, pathways, functions, and PPIs. For direct proteins and PPIs, the drug-associated sample includes the drug’s protein target set. For pathways and functions, the drug-associated sample encompasses those pathways and functions targeted by way of direct proteins, as described above. For direct proteins, pathways, functions, and PPIs, the disease-associated set entails the list of regulated genes, indirect proteins by PPIs, overrepresented pathways, and overrepresented functions, respectively. Convert p-values at each biological level to normalized scores by way of log transformation followed by normalization to the log-transformed p-value of the most significantly associated drug for the level of analysis in question. Figure 3a and 3b for further details.
11. To rank all annotated drugs against a given disease, combine level-specific normalized significance scores to a cumulative drug-association Z-scores using the weight configuration described in equation 2. These Z-scores may be subsequently ranked to judge the potential of association with the disease. See Figure 3c and 3d for details.

**Note**: these instructions pertain only to the procedure for gene expression-based association of drugs to diseases by way of *known* (experimentally-derived) drug-target associations. The DGE-NET platform also consists of an identical procedure based instead on drug-target interactions predicted by the proteochemometric TMFS method [17], as well as the separate and simpler construction of a drug-target-pathway-function-disease network. Both omitted features are described in the Methods.
